# The world within reach: An image database of reach-relevant environments

**DOI:** 10.1167/jov.21.7.14

**Published:** 2021-07-21

**Authors:** Emilie L. Josephs, Haoyun Zhao, Talia Konkle

**Affiliations:** 1 Department of Psychology, Harvard University, Cambridge, MA, USA

**Keywords:** reachspaces, image database, embodied perception

## Abstract

Near-scale spaces are a key component of our visual experience: Whether for work or for leisure, we spend much of our days immersed in, and acting upon, the world within reach. Here, we present the Reachspace Database, a novel stimulus set containing over 10,000 images depicting first person, motor-relevant views at an approximated reachable scale (hereafter “reachspaces”), which reflect the visual input that an agent would experience while performing a task with her hands. These images are divided into over 350 categories, based on a taxonomy we developed, which captures information relating to the identity of each reachspace, including the broader setting and room it is found in, the locus of interaction (e.g., kitchen counter, desk), and the specific action it affords. Summary analyses of the taxonomy labels in the database suggest a tight connection between activities and the spaces that support them: While a small number of rooms and interaction loci afford many diverse actions (e.g., workshops, tables), most reachspaces were relatively specialized, typically affording only one main activity (e.g., gas station pump, airplane cockpit, kitchen cutting board). Overall, this Reachspace Database represents a large sampling of reachable environments and provides a new resource to support behavioral and neural research into the visual representation of reach-relevant environments. The database is available for download on the Open Science Framework (osf.io/bfyxk/).

## Introduction

Reachable environments are the locus of most of our interactions with the physical world: From the desk where we type an email, to the kitchen counter where we prepare our coffee, to the workbench where we assemble the pieces of a new project, we frequently experience near-scale views of the world. Recent results suggest that the perceptual processing of reachable-scale environments may diverge from that of navigable-scale spaces and single objects (Josephs & Konkle, 2019, 2020). However, to date, work in this area has been limited by a lack of high-quality image databases featuring such views. Domains like object perception, scene perception, action understanding, face identification, number understanding, and more have all benefited greatly from the existence of such databases (Bainbridge et al., 2012; Brady et al., 2008; Deng et al., 2009; Hebart et al., 2019; Horst & Hout, 2016; Zhou et al., 2018). These databases provide a double benefit: They identify, label, and taxonomize the elements of visual experience, and they provide stimuli for psychological and neuroimaging experiments. Here, we introduce an image database of reachable-scale views, with the goal of accelerating research in the perceptual, cognitive, and neural processes that underlie our understanding of the near-scale world.

**Figure 1.**
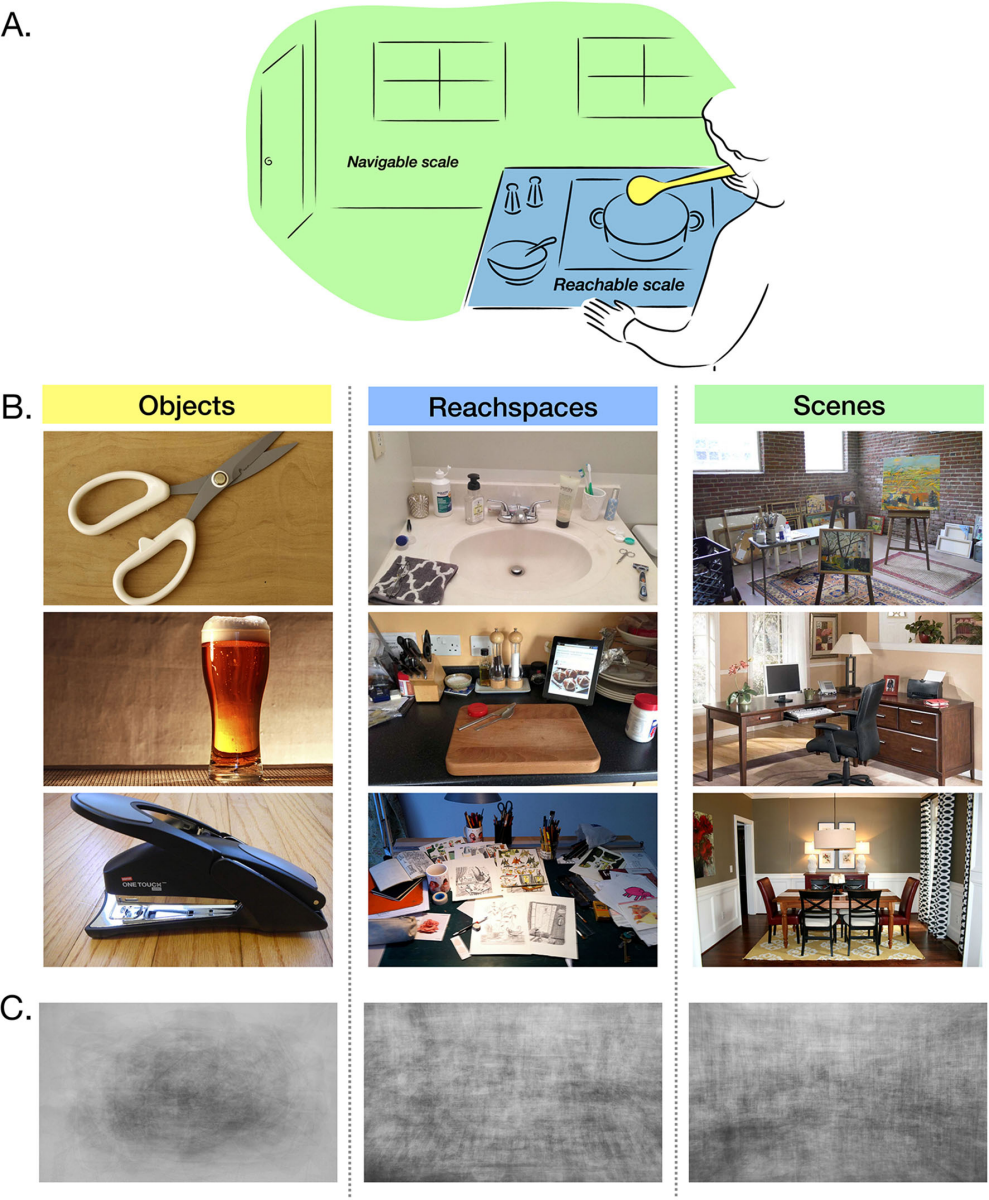
(A) Illustration of different scales of human experience. (B) Examples of common object, reachspace, and scene views. (C) Average of 50 object, reachspace, and scene images, respectively. This illustrates the differences in dominant orientation and clutter distributions across image types. Whole-image contrast was enhanced using Photoshop to increase visibility, but no other changes were applied.

We will use the term “reachspaces” to refer to these rich, reach-relevant spaces and formally define them as near-scale environments that support hand-based actions (Figure 1A). Reachspace views are first-person views of these spaces, taken from the perspective of an agent performing a task. They typically depict extended surfaces, oriented horizontally or vertically, populated with objects that support a common task. This also includes views of engineered structures that afford hand-based actions, such as control panels with buttons and knobs, or even large screens with touch-sensitive components. In all cases, the primary mode of behavior in these environments is for the agent to reach out and use their hands on the interactable units of the environment. To be clear, the term “reachspace” is not meant to refer to the parts of the world that are strictly within the arc of an agent's reach, or the three-dimensional spatial volume around the agent within which reach motions are made; terms such “peripersonal space,” “grasping space,” and “personal space” have been proposed for this; for reviews, see di Pellegrino and Ládavas (2015). Previc (1998), Cutting and Vishton (1995), and Grüsser (1983). Rather, it refers to the visual input corresponding to the environments that typically intersect with this volume, and where the primary behavior involves reaching.

Historically, vision science research has focused on mechanisms for processing views of singleton objects and navigable-scale spaces (i.e., “scenes”; for reviews, see Epstein, 2014; Groen et al., 2017), at the expense of the intermediate scale of reachable environments (cf. Henderson & Hollingworth, 1999). However, reachspaces are structurally and visually different from scenes and objects, with potential consequences for how they are perceptually represented.

Reachspaces generally feature a short receding surface plane, starting near the waist and extending a few feet away from the observer (Figure 1B), with higher visual clutter in the lower visual field (Previc, 1998), corresponding to objects resting on the surface. In contrast, scenes feature a large ground plane, starting from the feet and extending to the horizon, or until it intersects with large structures like walls, while singleton objects are convex and self-enclosed. These structural differences have consequences for the distribution of orientation and spatial frequency information in images depicting each of these scales (Figure 1B, C). Indeed, reachspace images were recently shown to have dissociable visual features from both scenes or objects, evident both in image-computable models and in perceptual signatures of visual search speed (Josephs & Konkle, 2019; Torralba & Oliva, 2002). Further, reachspace images activate a distinct network of regions in the visual system from both singleton objects and navigable-scale scenes (Josephs & Konkle, 2020). Thus, while reachspaces have not typically been studied as a separate class of input from scenes and objects, it is clear that they have interesting and unique structure, and models of human perception would benefit from a more specific understanding of their variety and visual structure.

Here we present the Reachspace Database, an image set containing over 10,000 images of reachable environments, collected from more than 350 different categories. We attempted to comprehensively sample reach-relevant environments, collecting images from many different settings and locations, with a focus toward ecologically valid views (i.e., close to those we experience when actively behaving in these environments). The database features a large variety of reachspace structure, layout, orientation, and function. This effort has two broad aims. The first is practical: Research in this area has been hindered in part by a scarcity of image stimuli. This database will provide a large-scale, highly varied pool of labeled images to fill this need. The second is theoretical: With such a large-scale sampling of reachspace environments, we can begin to develop an understanding of the relationships, hierarchies, and categories that organize them; our efforts reflect an attempt to develop a categorization scheme by which to group reachspace views. While this database was designed with cognitive and vision science research in mind, we believe it can also serve broader research efforts, from computer vision to user experience design.

## Method

There were two key challenges to building this database. First, there were no existing lists of common reach-relevant or task-relevant environments to guide our search for reachspace categories. Second, unlike objects and scenes, which have clear labels, reachspaces do not have unambiguous verbal labels. Instead, they are typically referred to using the name of the large object or structure that forms the base of the space (e.g., “desk,” “countertop,” “workbench,” “kiosk”) and generally lack a unique single-word label (e.g., the term “desk” does not distinguish between a desk with a computer workstation and a desk for crafting). We developed a procedure that allowed us to collect reachspace categories and assign them unambiguous names. First, we generated lists of reachspace categories from lists of activities, scenes, and objects; second, we collected images based on this list; third we created a reachspace taxonomy to describe these images; and fourth, we used this taxonomy to systematically guide further image collection.

### Identifying reachspace categories through common activities

**Figure 2.**
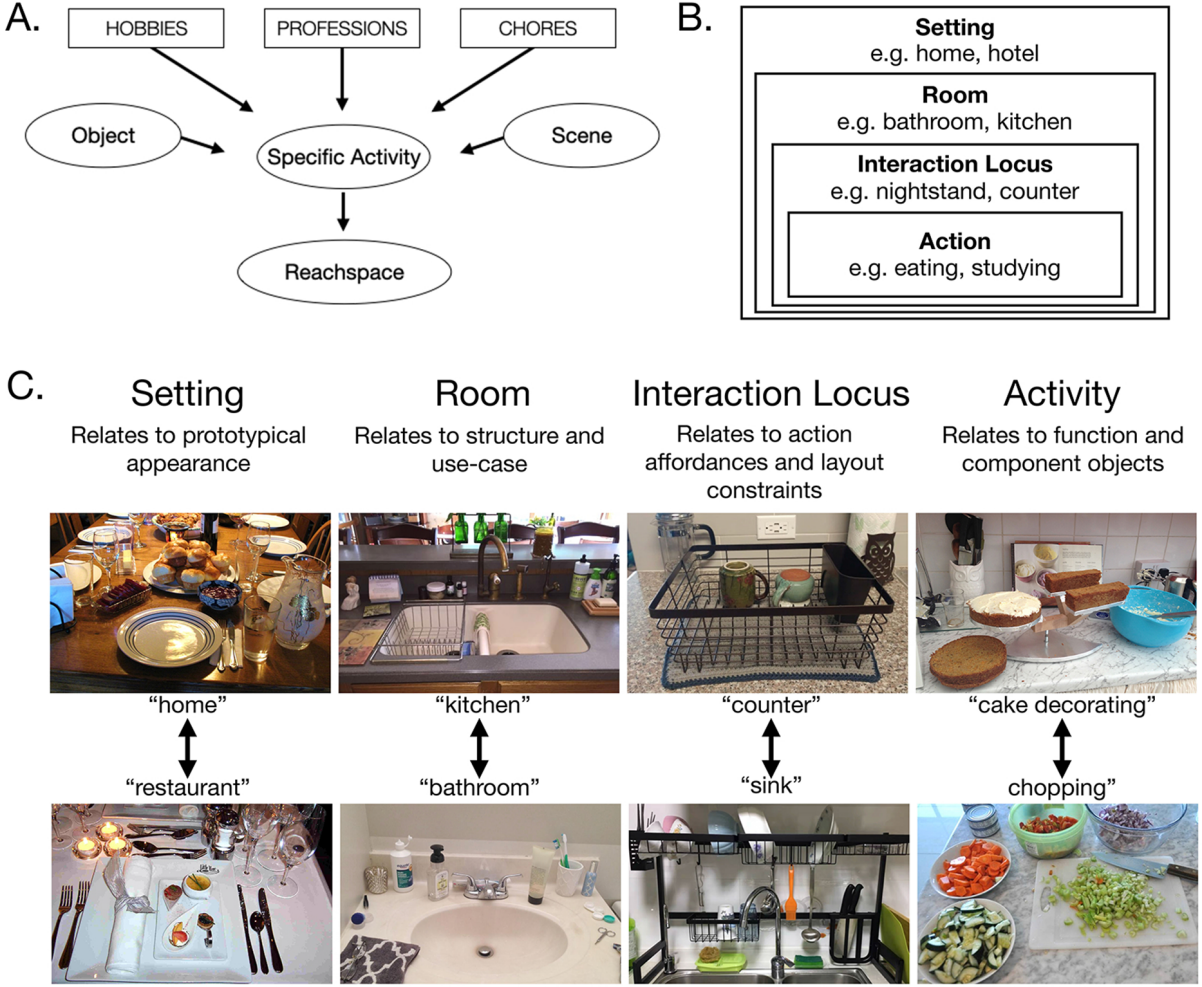
(A) Illustration of the procedure for generating reachspace categories: from lists of hobbies, chores, and professions, as well as common scenes and objects, we generated a list of hand-based activities, then identified the spaces that support those activities. (B) The naming convention adopted in this database employed a four-part taxonomy that labeled the setting, room, interaction locus, and activity relating to each reachspace. (C) For each level of the taxonomy, we provide examples of different labels and illustrate with images the kinds of differences that accompany category differences at the respective level.

Because of the lack of reachspace lists and labels, the initial generation of reachspace categories used an activity-centered approach (Figure 2A). A list of tasks and activities was collated from online lists of hobbies, professions, and household chores (this step was performed by authors ELJ and HZ, in conjunction with EH and JP listed in the Acknowledgments). Further activities were added in a more ad hoc manner, by starting from list of scene categories (e.g., “coffeeshop”) and generating the various separate tasks or activities that would be carried out in each (e.g., brewing the coffee, paying for the purchase, studying at a table, eating, retrieving a drink from the pickup counter). Additionally, some activities were generated by starting from objects (e.g., “wrench”) and generating tasks in which they are commonly used. Activities were not included in this list if they were not associated with near-space interactions (e.g., running), or if they involved hand–object interactions but are not associated with a stable reachable-scale environment (e.g., tennis). Thus, the output of this stage was a list of specific activities that people perform in reachable-scale environments, generated by considering tasks, places, and objects. Next, images of the spaces that support each activity were collected using online searches, as described below.

### Creating a taxonomic structure for cataloging the images

In order for the database to be convenient and searchable, reachspace images were divided into categories with unique and descriptive names. We developed a four-part naming convention (Figure 2B), in which the label for a given category is based on the setting it is in (the broader location type, e.g., hotel, home, office building, the outdoors), the room or site it occupies (e.g., dining room, conference room, campsite), the primary structure supporting the interaction with the environment (“interaction locus,” e.g., surfaces such as tables and shelves, or large interactable objects like control panels and digital kiosks), and the action it affords. For example, the picture in Figure 1B of a kitchen counter would be labeled home_kitchen_counter_chopping.

**Figure 3.**
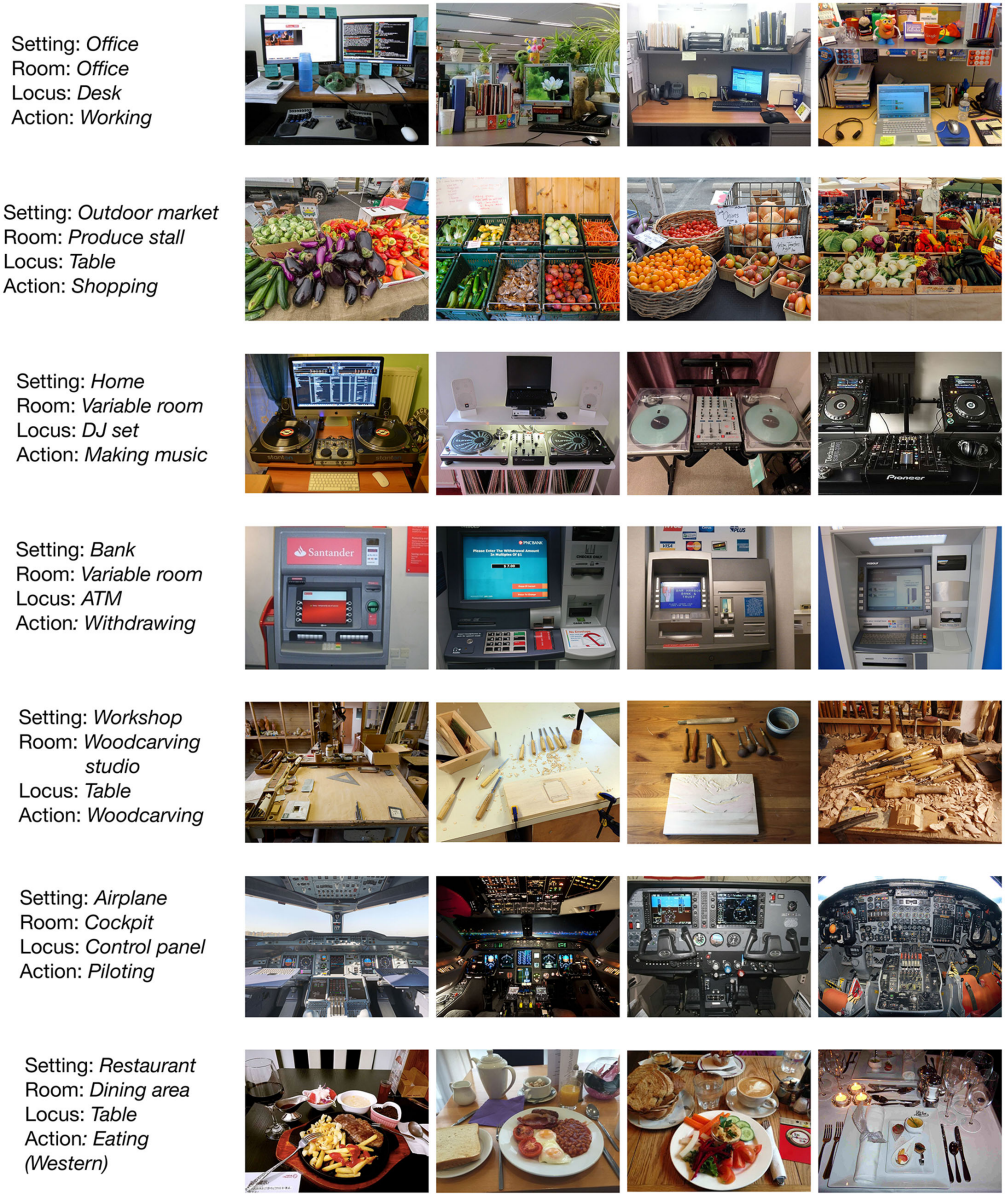
A selection of seven categories from the database, with four images per category, to illustrate the breadth and depth of the database and provide concrete examples of the naming convention.

This naming convention was developed because it provides a systematic and granular description of a given reachspace type, in a manner that informs expectations about the appearance, layout, components, and purpose of the space (Figure 2C). The setting label allows differentiation between reachspaces that are of a similar type but belong in different locales, which might have slightly different prototypical appearance because of their relationship to the broader setting (e.g., bathroom sink in a home vs. office vs. hotel). The room label is included because a given reachspace structure (e.g., a sink) will have a very different form and use-case depending on the room it belongs to (e.g., kitchen vs. bathroom vs. pottery studio). The interaction locus label recognizes that reachspaces will have different spatial constraints, visual appearances, and object layouts, depending on the surface they occupy (e.g., organizing your tools on a pegboard vs. in a toolbox). Finally, the action label allows differentiation between reachspaces that share a setting, room, and locus but support different actions by virtue of containing different objects (e.g., cake decorating vs. vegetable chopping on a kitchen counter). It also recognizes that reachspaces that differ in their setting, room, and interaction locus will nonetheless have common features if they afford the same activity (e.g., eating at a dining room table at home broadly involves similar actions and objects as eating on a picnic blanket at the beach). Overall, this naming convention was designed to highlight the components of a reachspace's identity that are most informative of its function and appearance (Figure 3).

There are some outliers or notable applications of this naming convention. First, some reachspaces are common to many different settings, with little visual or functional variation among them, such as elevator button panels and electrical breaker panels. Others are portable and thus are not strongly associated with any given setting, such as musical instrument cases or portable soldering benches. Such reachspaces were labeled “variableSetting” at the setting level. Second, some reachspace images did not have enough visual information or semantic constraints to be given a room label (e.g., children's toy sets can occupy any room of a house). Such reachspaces were labeled “variableRoom” at the room level. Finally, it was found that some reachspace categories had more granular divisions than could be captured with a four-level taxonomy. For example, the category restaurant_diningArea_table_eating does not distinguish between different kinds of restaurant place settings, which have different affordances and visual appearances (e.g., Western plates vs. South Asian thali). In such cases, a fifth label was appended, providing labels for the subcategories (e.g., restaurant_diningArea_table_eating_thali).

Labels at all levels were chosen to be general rather than specific. For example, while the individual actions afforded by a place setting at a restaurant are numerous (cutting steak, spooning soup, pouring wine, adding salt, etc.), we used the most general action label “eating” to encompass these. In some cases, the English word for the setting and room level of the taxonomy was the same (i.e., “office” can refer to an entire building housing a company, as well as a room within that building where an individual might work). In these cases, this word was repeated for each applicable level of the hierarchy. Category labels for this release of the database were generated primarily by author ELJ and vetted by author HZ and a second colleague not otherwise invested in the project (DJ, see Acknowledgments).

### Identifying additional reachspace categories using this taxonomic structure

In addition to organizing the images in the database, this taxonomy proved to be highly generative for discovering additional reachspace categories. Starting from a named category, different labels could be substituted at any level of the taxonomy to generate a new reachspace label. For example, from home_kitchen_counter_chopping, we could generate *restaurant*_kitchen_counter_chopping, home_kitchen_*table*_chopping, or home_kitchen_counter_*baking*. If the generated label corresponded to a real-world reachspace, images were collected for this category and included in the database following the procedure described below.

### Reachspace image collection

Images were collected for each of the identified categories using manual keyword searches in standard image search engines, from IP addresses in the United States and Hong Kong. Images were collected in the time period between September 2017 and October 2020. Since reachspace categories do not generally have specific names, the most effective search strategy for locating a given reachspace type was to append an activity name to the name of the furniture item that supports it (e.g., “crafting desk,” “illustrator's desk,” “work desk”). To find images depicting the whole reachable environment, with correct camera angle and framing, the following strategies were adopted: appending the first-person “my” to the search term (e.g., “my crafting desk”), adding adjectives such as “cluttered” or “organized” to the beginning, and appending “setup,” “layout,” or “display” to the end. To increase cultural diversity in the image set, we conducted searches on Baidu and Yahoo Japan in addition to Google and Bing, and used English, Chinese, and French search terms. Additionally, some reachspace images were original photographs taken by the experimenters. Finally, images were also found using the “Similar Image” features provided by many search engines.

All images in the database are real-world photographs (not CGI), saved in a jpeg format, and are in full color. Images were included if they met the following quality control criteria: (a) The image resolution is equal to or larger than 125 × 125 pixels (mean: 1,137 × 846); (b) the image depicts a view similar to what an observer would experience if they are standing or sitting in the space, actively engaging with it (depicted space is between approximately 2 and 4 feet in depth, camera position approximates an ecological viewing angle); and (c) the center of the space, where the bulk of the hand–object interactions takes place, is approximately centered in the image frame. Images were excluded if they contained clear views of people, hands, or faces; if they contained a large watermark (small watermarks on the edges of images were allowed); if they had clear filter effects; or if they depicted contrived layouts, such as those in staged advertising images. Images were occasionally cropped to better fit these criteria.

### Database location, availability, and format

All images are available for download in an OSF repository (https://osf.io/bfyxk/). The database consists of the images, divided into folders according to their categories. Image metadata is included in the form of a csv file, which lists the following metrics for each image: image name, setting-level label, room-level label, interaction locus label, action label, fifth-level label (if applicable), image resolution, and the number of other images in the same category. The database can also be previewed and explored at https://www.reachspacedatabase.com/.

## Results

**Figure 4.**
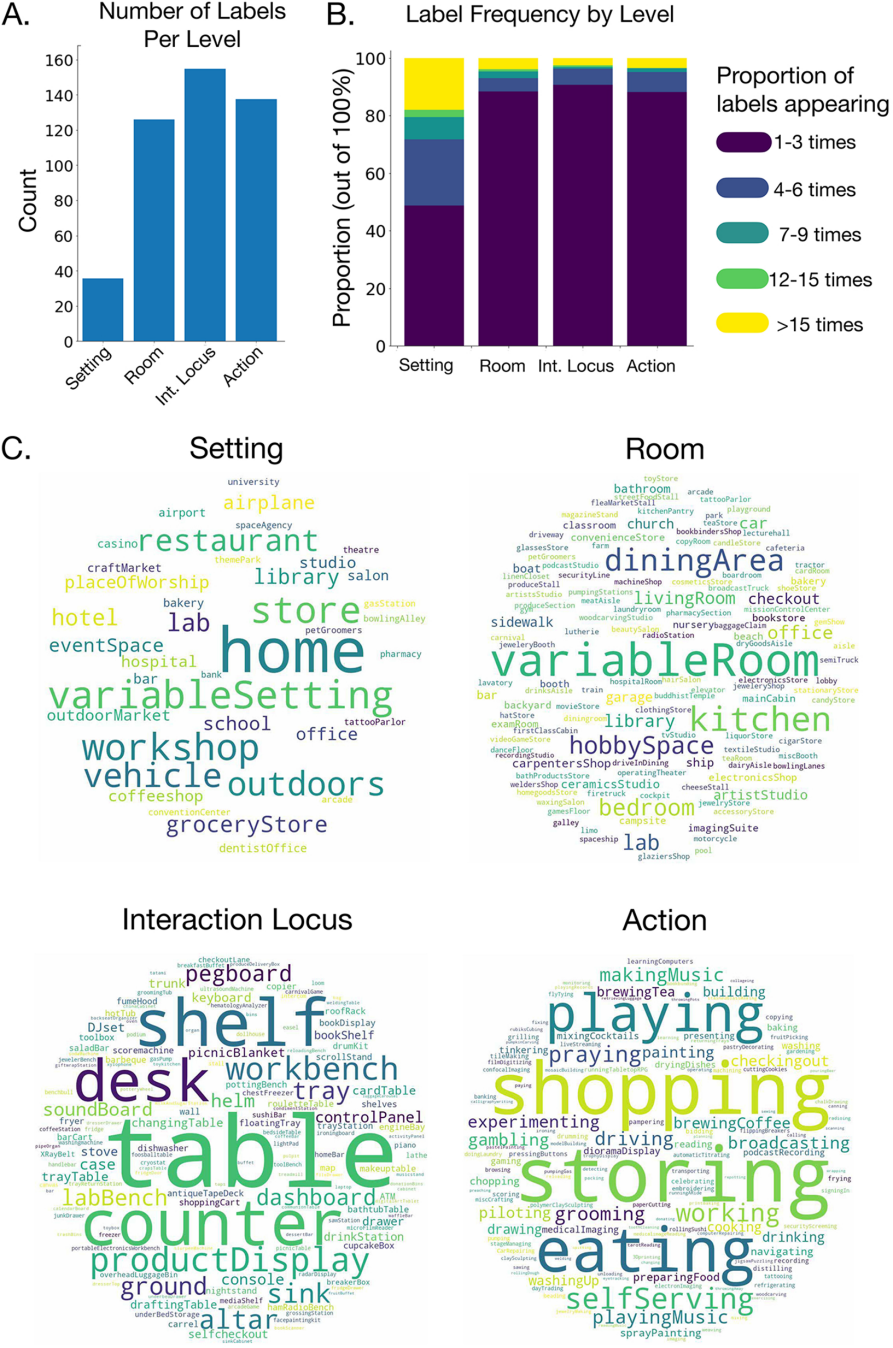
Metrics of the database. (A) Number of labels at each level of the taxonomy. (B) Frequency of labels across the levels of the hierarchy. The vast majority of labels appeared 1 to 3 times, although some labels appeared many times (> 15). (C) Overview of the labels appearing at each level of the hierarchy. Size of the word corresponds to the number of times it appears in category names at this level of the hierarchy. Note that “variableSetting” and “variableRoom” were used for reachspaces that do not have a canonical or clear association with a specific setting or room (see text).

The database contains a total of 11,276 images, drawn from 351 different categories (as of October 31, 2020, reflecting release 1.0). These categories are broadly sampled, reflecting the places where we eat, work, play, worship, create, play music, store items, and more (Figure 3). On average, there are 32 images per category (*SD* = 13), and the database only includes categories with a minimum of 10 images. Altogether, these categories represent 38 unique settings, 131 unique rooms, 161 unique interaction loci, and 143 unique actions (Figure 4A). Figure 4C depicts the unique labels at each level as word clouds, where the size of the text corresponds to the frequency of that label. Overall, the Reachspace Database (osf.io/bfyxk/) provides a broad and comprehensive sampling of the reach-relevant world.

In the next sections, we report the results of some summary analyses performed on the database, which provides a lens into the statistics of reachspaces in the world within the context of our sampling. We first asked how reachspace categories are distributed in the broader environment by counting how often each unique label appeared at the setting or room level of the taxonomy (Figure 4B), thus giving a rough census of the number of reachspaces associated with each context. Most settings were associated with only a small number of reachspaces (50.0% of setting labels only appeared one to three times), suggesting that a given setting generally supports a relatively limited set of activities. This is even more evident at the room level: 88.5% of rooms were associated with only one to three reachspaces, indicating high specificity in the activities conducted in a given room. However, these distributions have very long tails: 18.4% of settings and 3.4% of rooms were associated with greater than 15 reachspaces. Indeed, at the extreme, settings such as “home” and “workshop” and rooms such as “kitchen” were linked to more than 30 unique activities. Overall, this suggests that the statistics of reachspaces in the world are not uniformly distributed: While most locations support few (one to three) actions, other can be considered “hubs” supporting many actions.

Next, we examined how tightly the setting, room, and interaction locus together constrains the actions associated with a space. That is, given a space defined by a particular setting–room–locus chain, how many actions can be performed there? In the current database, 90.8% of setting–room–loci chains were associated with only 1 action, 5.3% with two actions, and only 3.9% with three or more actions. Thus, the setting, room, and interaction locus together strongly dictates what function a given reachspace is associated with. This is intuitive: Sitting at a table in the dining area of a restaurant is enough to strongly suggest that you are eating. As above, however, the shape of this distribution has a long tail, with a few setting–room–locus chains having associations with seven or more different actions (including home_kitchen_counter and home_hobbySpace_table). Thus, there may be an interesting division between reachspaces that support a single purpose and those that have high flexibility to remain general purpose.

**Figure 5.**
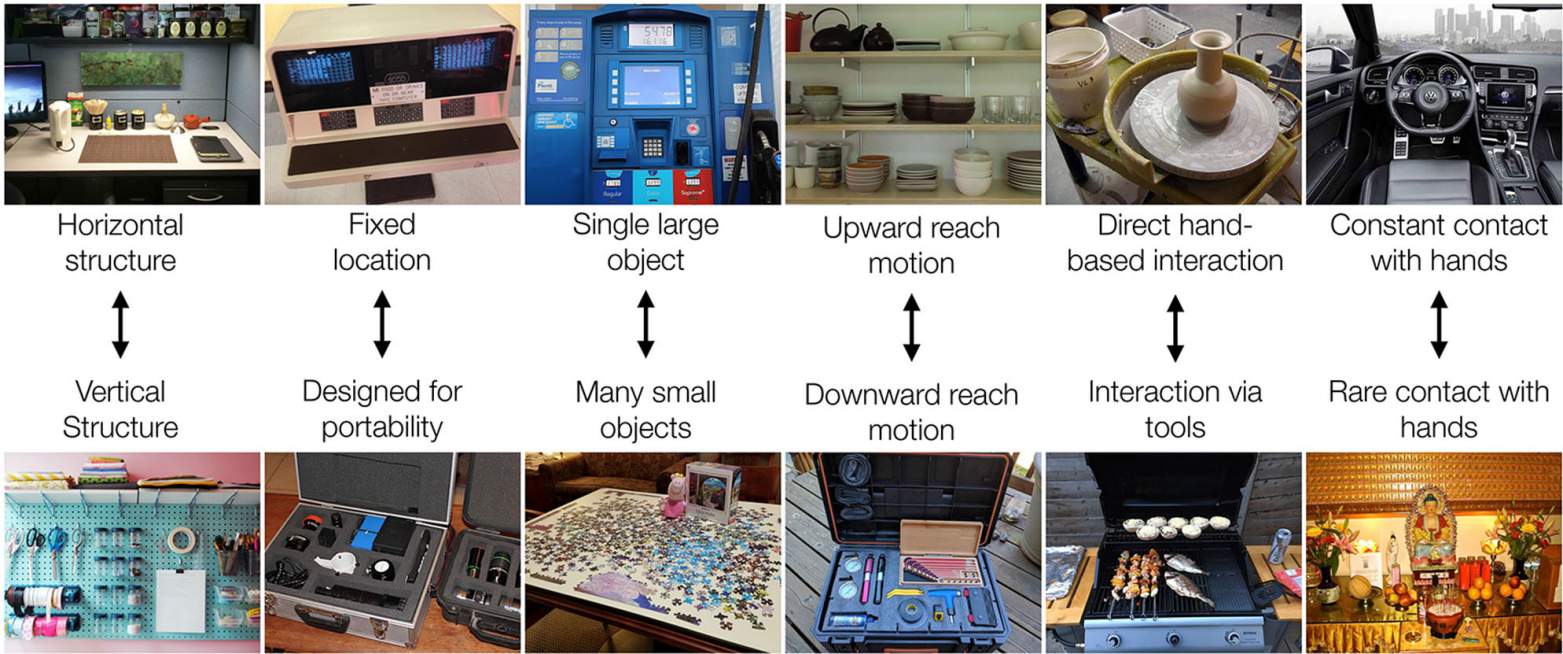
Some divisions observed among reachspaces during construction of the database.

Finally, in constructing the database, we observed that reachspaces could differ from each other on a number of dimensions that are not clearly linked to the taxonomy used to create the database (Figure 5). For example, we observed that (a) the primary spatial arrangement of objects in space might be horizontal or vertical; (b) the afforded reach motion might vary in its angle, from upward motion to horizontal motion to downward motion; (c) the reachspace might be static and tied to a specific location or be designed for portability; (d) might consist of a single large object or many small objects arrayed on a surface; (e) might afford direct action with the hands or require the intermediary of tools; and (f) might require constant hand-based interaction or be primarily engaged with using vision. This nonexhaustive list of properties is based on our observations while collecting the images; concurrent work is under way to measure the similarity structure among reachspace images in human behavioral judgments and to discover the dimensions underlying this structure using data-driven methods recently employed for objects (Hebart et al., 2020).

## Discussion

Here, we introduce the Reachspace Database, a database of images depicting the rich, near-scale views we experience when performing hand-based tasks and activities in the world (available at osf.io/bfyxk/). While many image databases exist for human and computer vision, this is the first, to our knowledge, that explicitly captures ecological views centered on a reachable scale.

**Figure 6.**
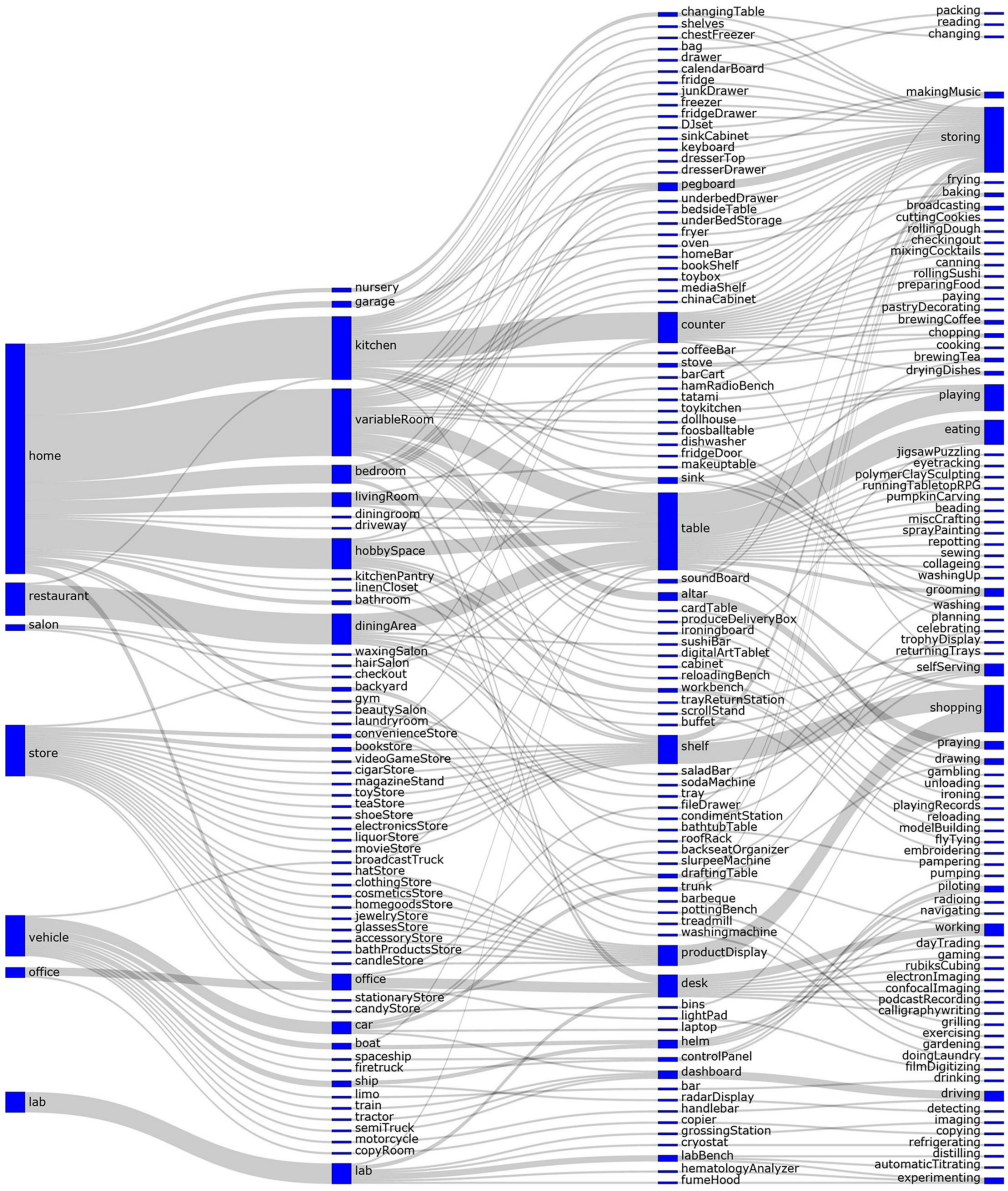
Illustration of the relationships among taxonomy level labels for 192 example categories, selected from seven example settings. Specifically, this figure demonstrates how the current taxonomy is not strictly hierarchical: The same room can exist in different settings, and the same interaction locus (e.g., table) can exist in many different rooms. Blue bars represent labels at each level of the taxonomy, and the size of the bars represents the number of times that label appears. Gray lines connect labels that together form a category name.

The act of collecting and cataloging reachspace images led to the development of a naming convention and taxonomy, which groups reachspaces based on the spaces they appear in and the action they afford. This system was selected because we found it was the most helpful for looking up reachspace images and informing expectations of their appearance, but we make no claims about whether it reflects how knowledge about reachspaces is organized in the mind. One interesting feature of this taxonomy is that it is not strictly hierarchical. While reachspaces are embedded in rooms, which are themselves embedded in broader environments, the relationship among these is not exclusive. For example, a kitchen can be found in a house, an office building, a hotel, or a restaurant. Thus, subordinate labels in this taxonomy are not exclusively nested under superordinate ones. This is illustrated in Figure 6, using a subset of categories. This complex structure suggests that a reachspace's specific identity is not adequately captured by any single level of the taxonomy and that its precise attributes will be related to its context as well as its function.

One possible alternative system would be to classify reachspaces based on their function or purpose, at different levels of abstraction. Function has been shown to be a salient property, which plays a large role in high-level judgments of similarity for objects, scenes, and actions (Greene et al., 2016; Rosch et al., 1976; Tversky, 1989). Under this scheme, a superordinate label might refer to the broad activity a reachspace affords (playing, working, doing art), with more specific activities appearing under each (playing chess vs. playing with Legos; typing vs. reading; painting vs. sculpting). However, reachspaces are related to locations in lawful ways, and this alternative would not account for the important differences we have highlighted that arise from location differences.

It is interesting to note that, in contrast to the highly specific four-part names we have given to reachspace categories, natural-language labels for reachspaces are relatively vague: We speak of returning to our “desk” after lunch or searching our “bench” for a particular tool. This raises the question: Why do reachspace categories lack distinct labels in natural language? One possibility is that terms of reference such as “desk” or “workbench” are actually relatively unambiguous in the context of a natural utterance, when they can be integrated with knowledge about where the speaker is located and what they are doing (Carroll, 1980; Olson, 1970). Future work is required to understand the rules by which active and descriptive language references these interaction spaces and how linguistic conventions and terms of reference vary across languages.

There are limitations with the database in its current iteration. First, while we have taken pains to present a comprehensive sample of reachspaces, this database is by no means a complete or representative survey. This image set is biased toward spaces that are more commonly photographed from a first-person perspective or posted online. Thus, reachspaces associated with many professions — such as industrial kitchens, firetruck steering wheels, or judges' benches — are not represented. Additionally, given the backgrounds of the researchers and the kinds of images that tend to be featured in Internet searches, our sample reflects a cultural context that is highly industrialized and largely Western (see Henrich et al., 2010). Our activity-based approach in generating reachspace categories encouraged broad sampling of professions, but we have likely under sampled the variety of reachspaces from domains we are less familiar with (e.g., manufacturing and machining shops). Thus, the number of reachspace categories here should not be interpreted as an estimate of the number of reachspace categories in the world. Second, the names we have given to the categories represent our attempts to make different categories distinguishable from each other at multiple levels (setting, action, etc.) but should not be taken to provide canonical names for reachspace categories. While we internally validated that each level of the label fit the images in the category, this was done on a small scale (i.e., agreement among three people; see Method section), and we have not broadly validated that these are the labels that are most commonly or naturally used in speech. Additionally, in choosing category names, we were limited to selecting only one label for each taxonomic level, so labels do not reflect the full richness of the language used to talk about these spaces (e.g., the kitchen reachspace that supports chopping could also be said to afford slicing, dicing, etc.). Future versions of the database would benefit from collecting a fuller sample of related terms. Altogether, these limitations indicate areas to focus on in future releases but do not stand in the way of the broader goal of fueling research into near-scale reach-relevant environments.

Overall, the goal of this database is to turn a spotlight onto the rich visual input that we experience during close-scale interactions with the world. Scene perception research has largely focused on large-scale views of the environment, and thus many of the insights from this area have centered on place recognition, the perception of navigational affordances, and how extended spatial layouts guide search, memory, and attention. It is not clear how these insights translate to reach-relevant spaces. We hope that this database will encourage research into reachspaces alongside navigable-scale scenes and will lead to more complete models of human perception at all scales.
